# Supplementary material for: Assessing Global Efforts in the Selection of Vertebrates as Umbrella Species for Conservation
Source: Biology (Basel). 2023 Mar 28;12(4):509. doi: 10.3390/biology12040509 (PMC10135637; doi:10.3390/biology12040509)
Supplement: Supplementary file 1 [file biology-12-00509-s001.zip › table S1-0304.pdf]

Table S1. A full list of the 242 scientific articles with umbrella species as study species published since 1984 to 2021, and terrestrial vertebrate species they recommended as umbrella species.

No.	Article	Published year	Common name	Scientific name	Class	Study country /region
1	De Vries 1995	1995	Alpine Ibex	*Capra ibex*	Mammalia	Europe
2	De Vries 1995	1995	Iberian Ibex	*Capra pyrenaica*	Mammalia	Europe
3	De Vries 1995	1995	European Roe deer	*Capreolus capreolus*	Mammalia	Europe
4	De Vries 1995	1995	Red Deer	*Cervus elaphus*	Mammalia	Europe
5	De Vries 1995	1995	Northern Chamois	*Rupicapra rupicapra*	Mammalia	Europe
6	De Vries 1995	1995	Wild Boar	*Sus scrofa*	Mammalia	Europe
7	Noss et al. 1996	1996	Brown Bear	*Ursus arctos*	Mammalia	Canada, US
8	Berger 1997	1997	Black Rhino	*Diceros bicornis*	Mammalia	Africa
9	Singh et al. 1997	1997	Lion-tailed Macaque	*Macaca silenus*	Mammalia	India
10	Martikainen et al. 1998	1998	White-backed Woodpecker	*Dendrocopos leucotos*	Aves	Finland, Russian Karelia
11	Savignac et al. 2000	2000	Pileated Woodpecker	*Hylatomus pileatus*	Aves	Canada
12	van Langevelde et al. 2000	2000	Eurasian Nuthatch	*Sitta europaea*	Aves	Netherland
13	Carroll et al. 2001	2001	Canada Lynx	*Lynx canadensis*	Mammalia	Canada, US
14	Carroll et al. 2001	2001	Brown Bear	*Ursus arctos*	Mammalia	Canada, US
15	Gerrard et al. 2001	2001	Kit Fox	*Vulpes macrotis*	Mammalia	US
16	Longepierre et al. 2001	2001	Hermann's Tortoise	*Testudo hermanni*	Reptilia	Europe
17	Loyn et al. 2001	2001	Powerful Owl	*Ninox strenua*	Aves	Australia
18	Loyn et al. 2001	2001	Greater Sooty-owl	*Tyto tenebricosa*	Aves	Australia
19	Poiani et al. 2001	2001	Greater Prairie-chicken	*Tympanuchus cupido*	Aves	US
20	Rubinoff 2001	2001	California Gnatcatcher	*Polioptila californica*	Aves	US
21	Snaith and Beazley 2002	2002	Moose	*Alces alces*	Mammalia	Canada
22	Suter et al. 2002	2002	Western Capercaillie	*Tetrao urogallus*	Aves	Switzerland
23	Borghesio and Ndang'ang'a 2003	2003	Fischer's Turaco	*Tauraco fischeri*	Aves	Tanzania
24	Kerley et al. 2003	2003	Blesbok	*Damaliscus pygargus*	Mammalia	South Africa
25	Kerley et al. 2003	2003	Mountain Zebra	*Equus zebra*	Mammalia	South Africa
26	Kerley et al. 2003	2003	Leopard	*Panthera pardus*	Mammalia	South Africa
27	Pakkala et al. 2003	2003	Western Capercaillie	*Tetrao urogallus*	Aves	Finland
28	Rubino and Hess 2003	2003	Barred Owl	*Strix varia*	Aves	US
29	Beazley and Cardinal 2004	2004	Golden Eagle	*Aquila chrysaetos*	Aves	Canada, US
30	Beazley and Cardinal 2004	2004	Upland Sandpiper	*Bartramia longicauda*	Aves	Canada, US

31	Beazley and Cardinal 2004	2004	Northern Harrier	*Circus hudsonius*	Aves	Canada, US
32	Beazley and Cardinal 2004	2004	Peregrine Falcon	*Falco peregrinus*	Aves	Canada, US
33	Beazley and Cardinal 2004	2004	Common Loon	*Gavia immer*	Aves	Canada, US
34	Beazley and Cardinal 2004	2004	Bald Eagle	*Haliaeetus leucocephalus*	Aves	Canada, US
35	Beazley and Cardinal 2004	2004	Reindeer	*Rangifer tarandus*	Mammalia	Canada, US
36	Cadi et al. 2004	2004	European Pond Turtle	*Emys orbicularis*	Reptilia	France
37	Chouteau 2004	2004	Coquerel's Coua	*Coua coquereli*	Aves	Madagascar
38	Comiskey et al. 2004	2004	Puma	*Puma concolor*	Mammalia	US
39	Jones et al. 2004	2004	Cerulean Warbler	*Setophaga cerulea*	Aves	Canada
40	Maciantowicz and Najbar 2004	2004	European Pond Turtle	*Emys orbicularis*	Reptilia	Poland
41	Nikolakaki 2004	2004	Common Redstart	*Phoenicurus phoenicurus*	Aves	England
42	Bifolchi and Lode 2005	2005	Eurasian Otter	*Lutra lutra*	Mammalia	North America
43	Matsuura et al. 2005	2005	Grey-faced Buzzard	*Butastur indicus*	Aves	Japan
44	Mildenstein et al. 2005	2005	Golden-capped Fruit Bat	*Acerodon jubatus*	Mammalia	Philippines
45	Mildenstein et al. 2005	2005	Large Flying-fox	*Pteropus vampyrus*	Mammalia	Philippines
46	Suorsa et al. 2005	2005	Eurasian Treecreeper	*Certhia familiaris*	Aves	Finland
47	Bani et al. 2006	2006	Short-toed Treecreeper	*Certhia brachydactyla*	Aves	Italy
48	Bani et al. 2006	2006	Marsh Tit	*Poecile palustris*	Aves	Italy
49	Bani et al. 2006	2006	Eurasian Nuthatch	*Sitta europaea*	Aves	Italy
50	Dunk et al. 2006	2006	Spotted Owl	*Strix occidentalis*	Aves	US
51	Hager et al. 2006	2006	Snapping Turtle	*Chelydra serpentina*	Reptilia	North America
52	Hager et al. 2006	2006	Painted Turtle	*Chrysemys picta*	Reptilia	North America
53	Hager et al. 2006	2006	Common Loon	*Gavia immer*	Aves	North America
54	Hager et al. 2006	2006	Bald Eagle	*Haliaeetus leucocephalus*	Aves	North America
55	Hager et al. 2006	2006	White-tailed Deer	*Odocoileus virginianus*	Mammalia	North America
56	Hager et al. 2006	2006	Osprey	*Pandion haliaetus*	Aves	North America
57	Hager et al. 2006	2006	Red Fox	*Vulpes vulpes*	Mammalia	North America
58	Koper and Schmiegelow 2006	2006	Northern Pintail	*Anas acuta*	Aves	Canada
59	Koper and Schmiegelow 2006	2006	Mallard	*Anas platyrhynchos*	Aves	Canada

60	Koper and Schmiegelow 2006	2006	Lesser Scaup	*Aythya affinis*	Aves	Canada
61	Koper and Schmiegelow 2006	2006	Gadwall	*Mareca strepera*	Aves	Canada
62	Koper and Schmiegelow 2006	2006	Northern shoveler	*Spatula clypeata*	Aves	Canada
63	Koper and Schmiegelow 2006	2006	Blue-winged Teal	*Spatula discors*	Aves	Canada
64	Moran-lopez et al. 2006	2006	Cinereous Vulture	*Aegypius monachus*	Aves	Spain
65	Ozaki et al. 2006	2006	Northern Goshawk	*Accipiter gentilis*	Aves	Japan
66	Rowland et al. 2006	2006	Sage Grouse	*Centrocercus urophasianus*	Aves	US
67	Sergio et al. 2006	2006	Northern Goshawk	*Accipiter gentilis*	Aves	Europe
68	Sergio et al. 2006	2006	Boreal Owl	*Aegolius funereus*	Aves	Europe
69	Sergio et al. 2006	2006	Northern Long-eared Owl	*Asio otus*	Aves	Europe
70	Sergio et al. 2006	2006	Eurasian Pygmy-owl	*Glaucidium passerinum*	Aves	Europe
71	Sergio et al. 2006	2006	Palawan Scops-owl	*Otus fuliginosus*	Aves	Europe
72	Sergio et al. 2006	2006	Tawny Owl	*Strix aluco*	Aves	Europe
73	Thorne et al. 2006	2006	Puma	*Puma concolor*	Mammalia	US
74	Castellon and Sieving 2007	2007	Black-throated Huet-huet	*Pteroptochos tarnii*	Aves	South America
75	Castellon and Sieving 2007	2007	Chucao Tapaculo	*Scelorchilus rubecula*	Aves	South America
76	Koper and Schmiegelow 2007	2007	Northern Pintail	*Anas acuta*	Aves	Canada
77	Koper and Schmiegelow 2007	2007	Mallard	*Anas platyrhynchos*	Aves	Canada
78	Koper and Schmiegelow 2007	2007	Lesser Scaup	*Aythya affinis*	Aves	Canada
79	Koper and Schmiegelow 2007	2007	Gadwall	*Mareca strepera*	Aves	Canada
80	Koper and Schmiegelow 2007	2007	Northern shoveler	*Spatula clypeata*	Aves	Canada
81	Koper and Schmiegelow 2007	2007	Blue-winged Teal	*Spatula discors*	Aves	Canada

82	Lynam et al. 2007	2007	Tiger	*Panthera tigris*	Mammalia	Malaysia
83	Bar-David et al. 2008	2008	Persian Fallow Deer	*Dama mesopotamica*	Mammalia	Israel
84	Dalerum et al. 2008	2008	Cheetah	*Acinonyx jubatus*	Mammalia	Africa
85	Dalerum et al. 2008	2008	Spotted Hyaena	*Crocuta crocuta*	Mammalia	Africa
86	Dalerum et al. 2008	2008	Striped Hyaena	*Hyaena hyaena*	Mammalia	Africa
87	Dalerum et al. 2008	2008	African Wild Dog	*Lycaon pictus*	Mammalia	Africa
88	Dalerum et al. 2008	2008	Lion	*Panthera leo*	Mammalia	Africa
89	Dalerum et al. 2008	2008	Leopard	*Panthera pardus*	Mammalia	Africa
90	Gallo-Reynoso et al. 2008	2008	Neotropical Otter	*Lontra longicaudis*	Mammalia	Mexico
91	Hurme et al. 2008	2008	Siberian Flying Squirrel	*Pteromys volans*	Mammalia	Europe
92	Keuroghlian and Eaton 2008	2008	White-lipped Peccary	*Tayassu pecari*	Mammalia	Brazil
93	Leech et al. 2008	2008	New Zealand Kaka	*Nestor meridionalis*	Aves	New Zealand
94	Mangas et al. 2008	2008	European Wildcat	*Felis silvestris*	Mammalia	Spain
95	Mangas et al. 2008	2008	Beech Marten	*Martes foina*	Mammalia	Spain
96	Mangas et al. 2008	2008	Eurasian Badger	*Meles meles*	Mammalia	Spain
97	Mangas et al. 2008	2008	Red Fox	*Vulpes vulpes*	Mammalia	Spain
98	Roberge et al. 2008	2008	White-backed Woodpecker	*Dendrocopos leucotos*	Aves	Sweden
99	Winchell and Doherty 2008	2008	California Gnatcatcher	*Polioptila californica*	Aves	US
100	Beier et al. 2009	2009	Puma	*Puma concolor*	Mammalia	US
101	Beier et al. 2009	2009	American Badger	*Taxidea taxus*	Mammalia	US
102	Beier et al. 2009	2009	Kit Fox	*Vulpes macrotis*	Mammalia	US
103	Ciudad et al. 2009	2009	Middle Spotted Woodpecker	*Leiopicus medius*	Aves	Spain
104	Grigione et al. 2009	2009	Jaguarundi	*Herpailurus yagouaroundi*	Mammalia	US, Mexico
105	Grigione et al. 2009	2009	Ocelot	*Leopardus pardalis*	Mammalia	US, Mexico
106	Grigione et al. 2009	2009	Jaguar	*Panthera onca*	Mammalia	US, Mexico
107	Mortelliti et al. 2009	2009	Edible Dormouse	*Glis glis*	Mammalia	Italy

108	Muller et al. 2009	2009	Middle Spotted Woodpecker	*Leiopicus medius*	Aves	Germany
109	Nekhay and Arriaza 2009	2009	Iberian Lynx	*Lynx pardinus*	Mammalia	Spain
110	Pruett et al. 2009	2009	Lesser Prairie-chicken	*Tympanuchus pallidicinctus*	Aves	US
111	Suazo et al. 2009	2009	Florida Scrub-jay	*Aphelocoma coerulescens*	Aves	US
112	Carroll et al. 2010	2010	Spotted Owl	*Strix occidentalis*	Aves	US
113	Hansbauer et al. 2010	2010	Rufous-breasted Leaftosser	*Sclerurus scansor*	Aves	Brazil
114	Poulin et al. 2010	2010	American Treecreeper	*Certhia americana*	Aves	Canada
115	Roberts et al. 2010	2010	Bobcat	*Lynx rufus*	Mammalia	US
116	Schlegel and Rupf 2010	2010	European Tree Frog	*Hyla arborea*	Amphibia	Switzerland
117	Tanneberger et al. 2010	2010	Aquatic Warbler	*Acrocephalus paludicola*	Aves	Pomerania
118	Barua 2011	2011	Jaguar	*Panthera onca*	Mammalia	Trans-continent
119	Barua 2011	2011	Tiger	*Panthera tigris*	Mammalia	Trans-continent
120	Barua 2011	2011	American Black Bear	*Ursus americanus*	Mammalia	Trans-continent
121	Barua 2011	2011	Brown Bear	*Ursus arctos*	Mammalia	Trans-continent
122	Blanco-Fontao et al. 2011	2011	Western Capercaillie	*Tetrao urogallus*	Aves	Spain
123	Davis et al. 2011	2011	Jaguar	*Panthera onca*	Mammalia	Belize
124	Edman et al. 2011	2011	White-backed Woodpecker	*Dendrocopos leucotos*	Aves	Poland
125	Epps et al. 2011	2011	African Savanna Elephant	*Loxodonta africana*	Mammalia	East Africa
126	Moreno-Opo et al. 2011	2011	Black Stork	*Ciconia nigra*	Aves	Spain
127	Onorato et al. 2011	2011	Puma	*Puma concolor*	Mammalia	US
128	Rozylowicz et al. 2011	2011	Grey Wolf	*Canis lupus*	Mammalia	Romania
129	Rozylowicz et al. 2011	2011	Eurasian Lynx	*Lynx lynx*	Mammalia	Romania
130	Rozylowicz et al. 2011	2011	Brown Bear	*Ursus arctos*	Mammalia	Romania
131	Sinsch et al. 2011	2011	Ahl's Reed Frog	*Hyperolius castaneus*	Amphibia	Rwanda, Congo
132	Stighall et al. 2011	2011	White-backed Woodpecker	*Dendrocopos leucotos*	Aves	Sweden

133	Stoffyn-Egli and Willison 2011	2011	American Beaver	*Castor canadensis*	Mammalia	US
134	Ucarli 2011	2011	Grey Wolf	*Canis lupus*	Mammalia	Turkey
135	Ucarli 2011	2011	Eurasian Lynx	*Lynx lynx*	Mammalia	Turkey
136	Ucarli 2011	2011	Brown Bear	*Ursus arctos*	Mammalia	Turkey
137	Burnham et al. 2012	2012	Clouded Leopard	*Neofelis nebulosa*	Mammalia	Trans-continent
138	Burnham et al. 2012	2012	Jaguar	*Panthera onca*	Mammalia	Trans-continent
139	Burnham et al. 2012	2012	Tiger	*Panthera tigris*	Mammalia	Trans-continent
140	Cushman and Landguth 2012	2012	Wolverine	*Gulo gulo*	Mammalia	US
141	Cushman and Landguth 2012	2012	American Marten	*Martes americana*	Mammalia	US
142	Cushman and Landguth 2012	2012	American Black Bear	*Ursus americanus*	Mammalia	US
143	Forrest et al. 2012	2012	Snow Leopard	*Panthera uncia*	Mammalia	Himalaya
144	Navedo and Garaita 2012	2012	Eurasian Spoonbill	*Platalea leucorodia*	Aves	Spain
145	Overdijk and Navedo 2012	2012	Eurasian Spoonbill	*Platalea leucorodia*	Aves	Spain
146	Ratnayeke and van Manen 2012	2012	Sloth Bear	*Melursus ursinus*	Mammalia	Sri Lanka
147	Brambilla et al. 2013	2013	Boreal Owl	*Aegolius funereus*	Aves	Italy
148	Brambilla et al. 2013	2013	Black Woodpecker	*Dryocopus martius*	Aves	Italy
149	Denoel et al. 2013	2013	Smooth Newt	*Lissotriton vulgaris*	Amphibia	Europe
150	Denoel et al. 2013	2013	Great Crested Newt	*Triturus cristatus*	Amphibia	Europe
151	Dinerstein et al. 2013	2013	Tiger	*Panthera tigris*	Mammalia	Trans-continent
152	McGranahan et al. 2013	2013	Greater Prairie-chicken	*Tympanuchus cupido*	Aves	US
153	Reza et al. 2013	2013	Sun Bear	*Helarctos malayanus*	Mammalia	Malaysia
154	Reza et al. 2013	2013	Tiger	*Panthera tigris*	Mammalia	Malaysia
155	Reza et al. 2013	2013	Sambar	*Rusa unicolor*	Mammalia	Malaysia
156	Reza et al. 2013	2013	Malay Tapir	*Tapirus indicus*	Mammalia	Malaysia
157	Shardlow and Hyatt 2013	2013	American Black Bear	*Ursus americanus*	Mammalia	Canada
158	Smith et al. 2013	2013	Northern Flying Squirrel	*Glaucomys sabrinus*	Mammalia	Canada
159	Stermin et al. 2013	2013	Western Water Rail	*Rallus aquaticus*	Aves	Romania
160	Alves et al. 2014	2014	Western Marsh-harrier	*Circus aeruginosus*	Aves	Europe
161	Blicharska et al. 2014	2014	White-backed Woodpecker	*Dendrocopos leucotos*	Aves	Sweden

162	Booms et al. 2014	2014	Short-eared Owl	*Asio flammeus*	Aves	North America
163	Breckheimer et al. 2014	2014	Red-cockaded Woodpecker	*Leuconotopicus borealis*	Aves	US
164	Copeland et al. 2014	2014	Sage Grouse	*Centrocercus urophasianus*	Aves	US
165	Gren et al. 2014	2014	White-backed Woodpecker	*Dendrocopos leucotos*	Aves	Sweden
166	Kaczensky et al. 2014	2014	Bactrian Camel	*Camelus ferus*	Mammalia	Mongolia
167	Kajtoch et al. 2014	2014	Goosander	*Mergus merganser*	Aves	Poland
168	Rosner et al. 2014	2014	Western Capercaillie	*Tetrao urogallus*	Aves	Europe
169	Tarjuelo et al. 2014	2014	Great Bustard	*Otis tarda*	Aves	Spain
170	Tarjuelo et al. 2014	2014	Little Bustard	*Tetrax tetrax*	Aves	Spain
171	Velo-Anton et al. 2014	2014	Nile Crocodile	*Crocodylus niloticus*	Reptilia	Mauritania
172	Winchell and Doherty 2014	2014	California Gnatcatcher	*Polioptila californica*	Aves	US
173	Bell et al. 2015	2015	White-backed Woodpecker	*Dendrocopos leucotos*	Aves	Sweden
174	Burnett and Roberts 2015	2015	Spotted Owl	*Strix occidentalis*	Aves	US
175	Carvalho et al. 2015	2015	Chimpanzee	*Pan troglodytes*	Mammalia	Guinea-Bissau
176	Chacon-Chaverri et al. 2015	2015	Green Turtle	*Chelonia mydas*	Reptilia	Costa Rica
177	Cristescu et al. 2015	2015	Brown Bear	*Ursus arctos*	Mammalia	Canada
178	Crosby et al. 2015	2015	Northern Bobwhite	*Colinus virginianus*	Aves	US
179	Dickman et al. 2015	2015	Sunda Clouded Leopard	*Neofelis diardi*	Mammalia	Trans-continent
180	Dickman et al. 2015	2015	Leopard	*Panthera pardus*	Mammalia	Trans-continent
181	Favilli et al. 2015	2015	Grey Wolf	*Canis lupus*	Mammalia	Carpathians
182	Favilli et al. 2015	2015	European Hare	*Lepus europaeus*	Mammalia	Carpathians
183	Favilli et al. 2015	2015	Eurasian Otter	*Lutra lutra*	Mammalia	Carpathians
184	Favilli et al. 2015	2015	Eurasian Lynx	*Lynx lynx*	Mammalia	Carpathians
185	Favilli et al. 2015	2015	Northern Chamois	*Rupicapra rupicapra*	Mammalia	Carpathians
186	Favilli et al. 2015	2015	Western Capercaillie	*Tetrao urogallus*	Aves	Carpathians
187	Favilli et al. 2015	2015	Brown Bear	*Ursus arctos*	Mammalia	Carpathians
188	Fedy et al. 2015	2015	Sage Grouse	*Centrocercus urophasianus*	Aves	US
189	Kiffner et al. 2015	2015	Common Wildebeest	*Connochaetes taurinus*	Mammalia	Tanzania
190	Kiffner et al. 2015	2015	Plains Zebra	*Equus quagga*	Mammalia	Tanzania

191	Litvaitis et al. 2015	2015	Bobcat	*Lynx rufus*	Mammalia	US
192	Lukacs et al. 2015	2015	Gunnison Grouse	*Centrocercus minimus*	Aves	US
193	Masse et al. 2015	2015	American Woodcock	*Scolopax minor*	Aves	US
194	McNew et al. 2015	2015	Greater Prairie-chicken	*Tympanuchus cupido*	Aves	US
195	Mikolas et al. 2015	2015	Western Capercaillie	*Tetrao urogallus*	Aves	Central Europe
196	Nekaris et al. 2015	2015	Red Slender Loris	*Loris tardigradus*	Mammalia	Sri Lanka
197	Nekaris et al. 2015	2015	Fishing Cat	*Prionailurus viverrinus*	Mammalia	Sri Lanka
198	Proctor et al. 2015	2015	Brown Bear	*Ursus arctos*	Mammalia	Canada, US
199	Puri et al. 2015	2015	Sloth Bear	*Melursus ursinus*	Mammalia	India
200	Santangeli et al. 2015	2015	White-tailed Sea-eagle	*Haliaeetus albicilla*	Aves	Finland
201	Viterbi et al. 2015	2015	Black Grouse	*Lyrurus tetrix*	Aves	Italy
202	Winder et al. 2015	2015	Greater Prairie-chicken	*Tympanuchus cupido*	Aves	US
203	Winder et al. 2015	2015	Lesser Prairie-chicken	*Tympanuchus pallidicinctus*	Aves	US
204	Alexander et al. 2016	2016	Snow Leopard	*Panthera uncia*	Mammalia	China
205	Bergner et al. 2016	2016	White-backed Woodpecker	*Dendrocopos leucotos*	Aves	Turkey
206	Bergner et al. 2016	2016	Syrian Woodpecker	*Dendrocopos syriacus*	Aves	Turkey
207	Bergner et al. 2016	2016	Middle Spotted Woodpecker	*Leiopicus medius*	Aves	Turkey
208	Bichet et al. 2016	2016	Reindeer	*Rangifer tarandus*	Mammalia	North America
209	Burgas et al. 2016	2016	Northern Goshawk	*Accipiter gentilis*	Aves	Finland
210	Burgas et al. 2016	2016	Ural Owl	*Strix uralensis*	Aves	Finland
211	Coates et al. 2016	2016	Sage Grouse	*Centrocercus urophasianus*	Aves	US
212	Collins and du Toit 2016	2016	Riverine Rabbit	*Bunolagus monticularis*	Mammalia	South Africa
213	Dahlgren et al. 2016a	2016	Sage Grouse	*Centrocercus urophasianus*	Aves	US
214	Dahlgren et al. 2016b	2016	Sage Grouse	*Centrocercus urophasianus*	Aves	US
215	Gangadharan et al. 2016	2016	Asian Elephant	*Elephas maximus*	Mammalia	India
216	Gangadharan et al. 2016	2016	Tiger	*Panthera tigris*	Mammalia	India
217	Higa et al. 2016	2016	Blakiston's Eagle-owl	*Bubo blakistoni*	Aves	Japan
218	Higa et al. 2016	2016	Red-crowned Crane	*Grus japonensis*	Aves	Japan
219	Ladin et al. 2016	2016	Wood Thrush	*Hylocichla mustelina*	Aves	US

220	Li and Pimm 2016	2016	Giant Panda	*Ailuropoda melanoleuca*	Mammalia	China
221	Maslo et al. 2016	2016	Piping Plover	*Charadrius melodus*	Aves	US
222	Maslo et al. 2016	2016	American Oystercatcher	*Haematopus palliatus*	Aves	US
223	McKellar et al. 2016	2016	Red-cockaded Woodpecker	*Leuconotopicus borealis*	Aves	US
224	Osipova and Sangermano 2016	2016	Jaguar	*Panthera onca*	Mammalia	Bolivia
225	Osipova and Sangermano 2016	2016	Southern Tamandua	*Tamandua tetradactyla*	Mammalia	Bolivia
226	Osipova and Sangermano 2016	2016	Lowland Tapir	*Tapirus terrestris*	Mammalia	Bolivia
227	Real et al. 2016	2016	Bonelli's Eagle	*Aquila fasciata*	Aves	Spain
228	Thornton et al. 2016	2016	Jaguar	*Panthera onca*	Mammalia	South and North America
229	Bradsworth et al. 2017	2017	Powerful Owl	*Ninox strenua*	Aves	Australia
230	Carlisle et al. 2017	2017	Sage Grouse	*Centrocercus urophasianus*	Aves	US
231	Duvall et al. 2017	2017	Sage Grouse	*Centrocercus urophasianus*	Aves	US
232	Fourcade et al. 2017	2017	Corncrake	*Crex crex*	Aves	France
233	Gamo and Beck 2017	2017	Sage Grouse	*Centrocercus urophasianus*	Aves	US
234	Johnson et al. 2017	2017	Gopher Tortoise	*Gopherus polyphemus*	Reptilia	US
235	Johnson et al. 2017	2017	Red-cockaded Woodpecker	*Leuconotopicus borealis*	Aves	US
236	Mikolas et al. 2017	2017	Western Capercaillie	*Tetrao urogallus*	Aves	Carpathians
237	Morelli et al. 2017	2017	Common Cuckoo	*Cuculus canorus*	Aves	Ukraine, Poland, Denmark
238	Tomecek et al. 2017	2017	Northern Bobwhite	*Colinus virginianus*	Aves	US
239	Wang et al. 2017	2017	Chinese Monal	*Lophophorus lhuysii*	Aves	China
240	Winder et al. 2017	2017	Greater Prairie-chicken	*Tympanuchus cupido*	Aves	US
241	Albert et al. 2018	2018	Tiger	*Panthera tigris*	Mammalia	Trans-continent
242	Carlisle et al. 2018a	2018	Sage Grouse	*Centrocercus urophasianus*	Aves	US
243	Carlisle et al. 2018b	2018	Sage Grouse	*Centrocercus urophasianus*	Aves	US

244	Cuadros-Casanova et al. 2018	2018	Sokoke Scops-owl	*Otus ireneae*	Aves	East Africa
245	Diniz et al. 2018	2018	Jaguar	*Panthera onca*	Mammalia	Brazil
246	Elliott and Johnson 2018	2018	Grasshopper Sparrow	*Ammodramus savannarum*	Aves	US
247	Figel et al. 2018	2018	Jaguar	*Panthera onca*	Mammalia	Central America
248	Gomez-Catasus et al. 2018	2018	Dupont's Lark	*Chersophilus duponti*	Aves	Spain
249	Hermes et al. 2018	2018	Ecuadorian Tapaculo	*Scytalopus robbinsi*	Aves	Ecuador
250	Hof and Hjalten 2018	2018	White-backed Woodpecker	*Dendrocopos leucotos*	Aves	Sweden
251	Hofman et al. 2018	2018	White-lipped Peccary	*Tayassu pecari*	Mammalia	Belize
252	Kittle et al. 2018	2018	Leopard	*Panthera pardus*	Mammalia	Sri Lanka
253	Macdonald et al. 2018	2018	Sunda Clouded Leopard	*Neofelis diardi*	Mammalia	Indonesia, Malaysia
254	Stuber and Fontaine 2018	2018	Grasshopper Sparrow	*Ammodramus savannarum*	Aves	US
255	Stuber and Fontaine 2018	2018	Lark Sparrow	*Chondestes grammacus*	Aves	US
256	Stuber and Fontaine 2018	2018	Northern Bobwhite	*Colinus virginianus*	Aves	US
257	Stuber and Fontaine 2018	2018	Common Pheasant	*Phasianus colchicus*	Aves	US
258	Stuber and Fontaine 2018	2018	Dickcissel	*Spiza Americana*	Aves	US
259	Stuber and Fontaine 2018	2018	Field Sparrow	*Spizella pusilla*	Aves	US
260	Stuber and Fontaine 2018	2018	Eastern Meadowlark	*Sturnella magna*	Aves	US
261	Stuber and Fontaine 2018	2018	Western Meadowlark	*Sturnella neglecta*	Aves	US
262	Vieira et al. 2018	2018	Black Skimmer	*Rynchops niger*	Aves	South and North America
263	Wang et al. 2018	2018	Giant Panda	*Ailuropoda melanoleuca*	Mammalia	China
264	Wei et al. 2018	2018	Giant Panda	*Ailuropoda melanoleuca*	Mammalia	China
265	Wiersma and Sleep 2018	2018	Reindeer	*Rangifer tarandus*	Mammalia	Canada
266	Banas et al. 2019	2019	Black Stork	*Ciconia nigra*	Aves	Poland
267	Brenner and McWilliams 2019	2019	American Woodcock	*Scolopax minor*	Aves	US

268	Crespo-Gascon and Guerrero-Casado 2019	2019	Spectacled Bear	*Tremarctos ornatus*	Mammalia	Andean ecoregions
269	Dinkins and Beck 2019	2019	Sage Grouse	*Centrocercus urophasianus*	Aves	US
270	Drever et al. 2019	2019	Reindeer	*Rangifer tarandus*	Mammalia	Canada
271	Ebenhoch et al. 2019	2019	Sage Grouse	*Centrocercus urophasianus*	Aves	US
272	Gorfol et al. 2019	2019	Western Barbastelle	*Barbastella barbastellus*	Mammalia	Hungary
273	Hawkes et al. 2019	2019	Eurasian Thick-knee	*Burhinus oedicnemus*	Aves	UK
274	Hof and Allen 2019	2019	Caucasian Grouse	*Lyrurus mlokosiewiczi*	Aves	Europe
275	Hof and Allen 2019	2019	Caucasian Snowcock	*Tetraogallus caucasicus*	Aves	Europe
276	Ionescu et al. 2019	2019	Black Stork	*Ciconia nigra*	Aves	Romania
277	Khosravi and Hemami 2019	2019	Cheetah	*Acinonyx jubatus*	Mammalia	Iran
278	Khosravi and Hemami 2019	2019	Sand Cat	*Felis margarita*	Mammalia	Iran
279	Khosravi and Hemami 2019	2019	Leopard	*Panthera pardus*	Mammalia	Iran
280	Klinga et al. 2019	2019	Western Capercaillie	*Tetrao urogallus*	Aves	Central Europe
281	Kramer et al. 2019	2019	American Woodcock	*Scolopax minor*	Aves	US
282	Kramer et al. 2019	2019	Golden-winged Warbler	*Vermivora chrysoptera*	Aves	US
283	Kumar et al. 2019	2019	Tiger	*Panthera tigris*	Mammalia	India
284	Lamb et al. 2019	2019	Long-tailed Duck	*Clangula hyemalis*	Aves	North America
285	Lee et al. 2019	2019	Yellow-throated Marten	*Martes flavigula*	Mammalia	South Korea
286	Li et al. 2019	2019	Asian Elephant	*Elephas maximus*	Mammalia	China
287	Macdonald et al. 2019	2019	Clouded Leopard	*Neofelis nebulosa*	Mammalia	Asia
288	Moran et al. 2019	2019	Jaguar	*Panthera onca*	Mammalia	Costa Rica
289	Pratt et al. 2019	2019	Sage Grouse	*Centrocercus urophasianus*	Aves	US
290	Puverel et al. 2019	2019	Black Woodpecker	*Dryocopus martius*	Aves	France
291	Runge et al. 2019	2019	Gunnison Grouse	*Centrocercus minimus*	Aves	US
292	Runge et al. 2019	2019	Sage Grouse	*Centrocercus urophasianus*	Aves	US
293	Shi et al. 2019	2019	Giant Panda	*Ailuropoda melanoleuca*	Mammalia	China
294	Shi et al. 2019	2019	Red Panda	*Ailurus fulgens*	Mammalia	China

295	Shi et al. 2019	2019	Chinese Giant Salamander	*Andrias davidianus*	Amphibia	China
296	Shi et al. 2019	2019	Golden Eagle	*Aquila chrysaetos*	Aves	China
297	Shi et al. 2019	2019	Baer's Pochard	*Aythya baeri*	Aves	China
298	Shi et al. 2019	2019	Takin	*Budorcas taxicolor*	Mammalia	China
299	Shi et al. 2019	2019	Chinese Three-striped Box Turtle	*Cuora trifasciata*	Reptilia	China
300	Shi et al. 2019	2019	Yellow-breasted Bunting	*Emberiza aureola*	Aves	China
301	Shi et al. 2019	2019	Bearded Vulture	*Gypaetus barbatus*	Aves	China
302	Shi et al. 2019	2019	Chinese Monal	*Lophophorus lhuysii*	Aves	China
303	Shi et al. 2019	2019	Forest Musk Deer	*Moschus berezovskii*	Mammalia	China
304	Shi et al. 2019	2019	Red Goral	*Naemorhedus baileyi*	Mammalia	China
305	Shi et al. 2019	2019	Clouded Leopard	*Neofelis nebulosa*	Mammalia	China
306	Shi et al. 2019	2019	Bengal Slow Loris	*Nycticebus bengalensis*	Mammalia	China
307	Shi et al. 2019	2019	Pygmy Slow Loris	*Nycticebus pygmaeus*	Mammalia	China
308	Shi et al. 2019	2019	Tiger	*Panthera tigris*	Mammalia	China
309	Shi et al. 2019	2019	Snow Leopard	*Panthera uncia*	Mammalia	China
310	Shi et al. 2019	2019	Golden Snub-nosed Monkey	*Rhinopithecus roxellana*	Mammalia	China
311	Shi et al. 2019	2019	Cabot's Tragopan	*Tragopan caboti*	Aves	China
312	Sibarani et al. 2019	2019	Sumatran Rhinoceros	*Dicerorhinus sumatrensis*	Mammalia	Indonesia
313	Sibarani et al. 2019	2019	Asian Elephant	*Elephas maximus*	Mammalia	Indonesia
314	Sibarani et al. 2019	2019	Tiger	*Panthera tigris*	Mammalia	Indonesia
315	Sibarani et al. 2019	2019	Sumatran Orangutan	*Pongo abelii*	Mammalia	Indonesia
316	Sitzia et al. 2019	2019	Western Capercaillie	*Tetrao urogallus*	Aves	Alps
317	Smith et al. 2019	2019	Sage Grouse	*Centrocercus urophasianus*	Aves	US
318	Stewart et al. 2019	2019	Blunt-nosed Leopard Lizard	*Gambelia sila*	Reptilia	US
319	Timmer et al. 2019	2019	Sage Grouse	*Centrocercus urophasianus*	Aves	US
320	Agha et al. 2020	2020	Sage Grouse	*Centrocercus urophasianus*	Aves	US
321	Agha et al. 2020	2020	Mojave Desert Tortoise	*Gopherus agassizii*	Reptilia	US
322	Allen et al. 2020	2020	Tiger	*Panthera tigris*	Mammalia	Indonesia
323	Ashrafzadeh et al. 2020	2020	Cheetah	*Acinonyx jubatus*	Mammalia	Iran
324	Ashrafzadeh et al. 2020	2020	Caracal	*Caracal caracal*	Mammalia	Iran
325	Ashrafzadeh et al. 2020	2020	Afro-Asiatic Wildcat	*Felis lybica*	Mammalia	Iran

326	Ashrafzadeh et al. 2020	2020	Eurasian Lynx	*Lynx lynx*	Mammalia	Iran
327	Ashrafzadeh et al. 2020	2020	Pallas's Cat	*Otocolobus manul*	Mammalia	Iran
328	Ashrafzadeh et al. 2020	2020	Leopard	*Panthera pardus*	Mammalia	Iran
329	Barlow et al. 2020	2020	Sage Grouse	*Centrocercus urophasianus*	Aves	US
330	Brennan et al. 2020	2020	African Savanna Elephant	*Loxodonta africana*	Mammalia	Botswana, Namibia, Zambia, Zimbabwe
331	Burivalova et al. 2020	2020	Sunda Clouded Leopard	*Neofelis diardi*	Mammalia	Indonesia
332	Burivalova et al. 2020	2020	Bornean Orangutan	*Pongo pygmaeus*	Mammalia	Indonesia
333	Carlisle and Chalfoun, 2020	2020	Sage Grouse	*Centrocercus urophasianus*	Aves	US
334	Carter et al. 2020	2020	Tiger	*Panthera tigris*	Mammalia	Asia
335	Drake et al. 2020	2020	Northern Myotis	*Myotis septentrionalis*	Mammalia	US
336	Drake et al. 2020	2020	Eastern Pipistrelle	*Perimyotis subflavus*	Mammalia	US
337	Freire Filho and Palmeirim, 2020	2020	Maranhão Red-handed Howler Monkey	*Alouatta ululata*	Mammalia	Brazil
338	Hamalainen et al. 2020	2020	White-backed Woodpecker	*Dendrocopos leucotos*	Aves	Finland
339	Hardy et al., 2020	2020	Greater Prairie-chicken	*Tympanuchus cupido*	Aves	US
340	Herrera-Sanchez et al. 2020	2020	Cuvier's Gazelle	*Gazella cuvieri*	Mammalia	North Africa
341	Klinga et al. 2020	2020	Western Capercaillie	*Tetrao urogallus*	Aves	Europe
342	Li et al. 2020	2020	Giant Panda	*Ailuropoda melanoleuca*	Mammalia	China
343	Linero et al., 2020	2020	Common Woolly Monkey	*Lagothrix lagothricha*	Mammalia	Amazon and Andes
344	Mena et al. 2020	2020	Jaguar	*Panthera onca*	Mammalia	Colombia, Ecuador, Peru
345	Natsukawa et al. 2020	2020	Northern Goshawk	*Accipiter gentilis*	Aves	Japan
346	Ortiz-Urbina et al. 2020	2020	Cinereous Vulture	*Aegypius monachus*	Aves	Spain
347	Petersen et al., 2020	2020	Clouded Leopard	*Neofelis nebulosa*	Mammalia	Southeast Asia
348	Pilliod et al. 2020	2020	Sage Grouse	*Centrocercus urophasianus*	Aves	US
349	Ruiz-Garcia et al. 2020	2020	Spectacled Bear	*Tremarctos ornatus*	Mammalia	Ecuador

350	Shen et al. 2020	2020	Giant Panda	*Ailuropoda melanoleuca*	Mammalia	China
351	Ward et al. 2020	2020	Regent Honeyeater	*Anthochaera phrygia*	Aves	Australia
352	Ward et al. 2020	2020	Australasian Bittern	*Botaurus poiciloptilus*	Aves	Australia
353	Ward et al. 2020	2020	Red Goshawk	*Erythrotriorchis radiatus*	Aves	Australia
354	Ward et al. 2020	2020	Far Eastern Curlew	*Numenius madagascariensis*	Aves	Australia
355	Ward et al. 2020	2020	Koala	*Phascolarctos cinereus*	Mammalia	Australia
356	Xu et al. 2020	2020	Chinese Monal	*Lophophorus lhuysii*	Aves	China
357	Andesner et al., 2021	2021	Western Capercaillie	*Tetrao urogallus*	Aves	Austria
358	Barichievy et al., 2021	2021	Black Rhino	*Diceros bicornis*	Mammalia	Kenya
359	Beranek et al., 2021	2021	Golden Bell Frog	*Litoria aurea*	Amphibia	Australia
360	Buffum et al., 2021	2021	American Woodcock	*Scolopax minor*	Aves	US
361	Curveira-Santos et al., 2021	2021	Lion	*Panthera leo*	Mammalia	South Africa
362	Dai et al., 2021	2021	Brown bear	*Ursus arctos*	Mammalia	Hindu Kush Himalayan
363	Kim et al., 2021	2021	White-naped Crane	*Grus vipio*	Aves	Korean DMZ
364	Lohmus et al., 2021	2021	Black Stork	*Ciconia nigra*	Aves	Estonia
365	Mizsei et al., 2021	2021	Greek Meadow Viper	*Vipera graeca*	Reptilia	Greece/Albania
366	Mukherjee et al., 2021	2021	Brown Bear	*Ursus arctos*	Mammalia	Himalayan region
367	Penjor et al., 2021	2021	Mainland Serow	*Capricornis sumatraensis*	Mammalia	Bhutan
368	Penjor et al., 2021	2021	Asiatic Golden Cat	*Catopuma temminckii*	Mammalia	Bhutan
369	Penjor et al., 2021	2021	Southern Red Muntjac	*Muntiacus muntjak*	Mammalia	Bhutan
370	Penjor et al., 2021	2021	Sambar	*Rusa unicolor*	Mammalia	Bhutan
371	Penjor et al., 2021	2021	Wild Boar	*Sus scrofa*	Mammalia	Bhutan
372	Rose et al., 2021	2021	Sage Grouse	*Centrocercus urophasianus*	Aves	Canada
373	Rosenblatt et al., 2021	2021	Northern Bobwhite	*Colinus virginianus*	Aves	US
374	Singer et al., 2021	2021	Bechstein's Myotis	*Myotis bechsteinii*	Mammalia	Germany
375	Smith et al., 2021	2021	Sage Grouse	*Centrocercus urophasianus*	Aves	US
376	Wang et al., 2021	2021	Giant Panda	*Ailuropoda melanoleuca*	Mammalia	China
377	Westekemper et al., 2021	2021	European Wildcat	*Felis silvestris*	Mammalia	Germany
378	Xu et al., 2021	2021	Scaly-sided Merganser	*Mergus squamatus*	Aves	China
379	Zhao et al., 2021	2021	Giant Panda	*Ailuropoda melanoleuca*	Mammalia	China
